# Community state shifts driven by total carbon availability over resource complexity in a synthetic microbial community

**DOI:** 10.1093/ismeco/ycag149

**Published:** 2026-05-29

**Authors:** Anna M Bischofberger, Johannes Cairns, Inga-Katariina Aapalampi, Sanna Pausio, Meri Lindqvist, Ville Mustonen, Teppo Hiltunen

**Affiliations:** Department of Biology, University of Turku, Turku, Turku 20014, Finland; Department of Biology, University of Turku, Turku, Turku 20014, Finland; Department of Experimental Medical Science, SciLifeLab, Lund University, Lund 223 62, Sweden; Turku Collegium for Science, Medicine and Technology, University of Turku, Turku 20014, Finland; Department of Computer Science, Department of Organismal and Evolutionary Biology, University of Helsinki, Helsinki 00014, Finland; Helsinki Institute of Sustainability Science (HELSUS), University of Helsinki, Helsinki 00014, Finland; Department of Biology, University of Turku, Turku, Turku 20014, Finland; Department of Biology, University of Turku, Turku, Turku 20014, Finland; Department of Biology, University of Turku, Turku, Turku 20014, Finland; Department of Computer Science, Department of Organismal and Evolutionary Biology, University of Helsinki, Helsinki 00014, Finland; Department of Biology, University of Turku, Turku, Turku 20014, Finland

**Keywords:** microbial ecology, experimental ecology, synthetic communities, community assembly, regime shift, resource gradient, carbon availability, resource complexity

## Abstract

Even though complex microbial communities are ubiquitous and provide essential services for natural and human-associated ecosystems, our knowledge about their assembly and dynamics is incomplete. There is an ongoing debate about whether the behavior of complex communities can be predicted from the outcome of pairwise competition of species, and whether communities reach alternative stable states depending on the level and complexity of resources provided for growth. To estimate the effect of two resource gradients, total carbon availability and resource complexity, on the compositional dynamics of a microbial community, we conducted a 16-day serial passage experiment, transferring a 16-species synthetic community in 96 different resource environments. We observed that although both resource dimensions influenced community composition, total carbon exerted a considerably larger effect. Additionally, we saw strong, discrete community state shifts along the total carbon gradient, a feature not observed for the resource complexity gradient. Using monoculture assays, we identified lag phase duration as the dominant predictor of competitive success at carbon extremes, with maximum growth rate increasing in importance as lag times converged. Total carbon availability thus structured community state transitions and regulated which growth trait governed competitive sorting. These results suggest the importance of total carbon level over resource complexity and identifying dominant species for the quest to successfully manage, maintain, and manipulate complex microbial communities.

## Introduction

Microorganisms exist in diverse, often complex communities. We depend on the functioning of these communities not only for the services they provide in natural ecosystems [1–3] but also for maintaining human health [4] and a broad variety of applications such as food production, wastewater treatment, and environmental bioremediation [5–7]. Identifying the mechanisms that govern microbial community assembly and dynamics and the conditions under which they are valid is thus crucial. Moreover, although high-throughput sequencing has revealed immense microbial diversity across environments, fundamental processes determining species co-existence, dominance, and exclusion are still incompletely understood.

Classical ecological theory predicts that community diversity is constrained by resource availability. The competitive exclusion principle states that the number of species able to co-exist is limited by the amount of resources, implying that increases in resource quantity or diversity should promote species richness [8, 9]. In microbial systems, however, community outcomes can deviate from such simple expectations. For example, microbial competition is not limited to passive depletion of shared resources, but can also involve interference mechanisms such as antimicrobial production, contact-dependent killing, biofilm exclusion, or exploitation of costly public goods [10]. In addition, mixed-resource environments may not behave additively: (1) nutrient pairs can interact so that one resource dominates community assembly, as shown for sugar–acid combinations where communities became more similar to sugar-grown communities than predicted from single-resource outcomes [11]; or (2) a combination of multiple carbon sources can enhance the growth of a bacterial community to levels higher than expected from growth observed in the individual constituent carbons [12].

Metabolic cross-feeding can also cause community behavior to deviate from simple expectations. By converting primary substrates into secondary metabolites that can be exploited by other taxa, cross-feeding effectively expands niche space even when communities are supplied with a single resource [13, 14]. Experimental studies have demonstrated that such metabolic interactions can generate stable multispecies communities and reproducible assembly patterns [15]. These dynamics extend simple resource partitioning frameworks by introducing cross-feeding networks analogous to trophic interactions in classical ecological theory.

Beyond resource quantity, the metabolic complexity, here referring to the diversity and heterogeneity, of available substrates can strongly influence community structure. Silverstein *et al.* [16] proposed the divergence-complexity hypothesis based on the principles that “metabolic diversity begets [microbial] diversity” and “[metabolic] diversity begets divergence [of microbial communities].” By culturing soil microbial samples with differing initial community composition at *t*_*0*_ in increasingly complex media formulations, they showed how metabolically complex resources provide a greater diversity of available niches, leading to increased divergence in community composition. This effect arises because complex substrates are degraded hierarchically into simpler compounds, generating metabolic by-products that enable cross-feeding interactions and support a broader set of specialist taxa. Other studies support this hypothesis in both natural and synthetic communities [17, 18]. Faced with ever greater disruption of natural environments due to human activity, it is essential to further our understanding of the dynamics behind regime shifts in and the resilience of microbial communities [19].

Co-existence may also be promoted in microbial communities through variation in life-history traits. Though known to modulate microbial competition, they remain underintegrated into community assembly frameworks. In particular, differences in maximal growth rate and/or lag-phase duration can critically influence competitive outcomes. The lag phase, a shift in metabolism from fermentation to respiration [20], reflects active physiological adjustment to novel environments and varies among species, environmental conditions, and even among individual cells [21–23]. Using donor stool communities and reconstituted synthetic versions of those communities, Aranda-Dìaz *et al.* [24] showed that removing the fastest-growing, initially dominant strains results in the second fastest strain reaching the highest abundance. The most successful strains exhibited a combination of high growth rate and short lag phase. Others have provided evidence, that in batch and serial-transfer systems, shorter lag phases alone can confer priority effects, allowing for early resource capture that may outweigh differences in maximal growth rate [25, 26]. Experimental evidence indicates that such priority effects can generate historical contingency and alternative community states [27]. However, growing evidence suggests that the outcomes of such community culturing depend not only on species’ life history traits but also on environmental and procedural factors [28–31].

Environmental conditions shape microbial community dynamics along multiple axes, including resource availability, life-history differences, and environmental stress. While this study focuses primarily on resource availability and life-history traits, environmental stress provides a complementary framework for how species interactions can shift along nutrient gradients. The stress-gradient hypothesis (SGH), originally developed in plant ecology, predicts that competition dominates under benign conditions, whereas facilitation becomes more important under increasing stress, often producing hump-shaped diversity patterns [32, 33]: at high environmental stress, the conditions are so harsh that only the hardiest and best adapted species can survive; in the absence of environmental stressors, organisms are able to invest resources in their competitive abilities, driving less-competitive species into extinction. Similar dynamics have been observed in microbial communities, where nutrient limitation or other stressors can shift interactions from competition toward facilitation [34–38].

Though recent advances in sequencing have helped elucidate the high diversity of microbial communities in different conditions and the availability of high-power computing allows for the quick processing of large datasets [39–41], there are still gaps in our ability to predict long-term community outcomes in changing environments. While there is evidence from some experiments that the complexity of community dynamics can be reduced to pairwise competition of species [42], there are other studies that highlight the importance of higher-order interactions for the dynamics of multispecies communities [43].

Here, we hypothesized that increasing resource complexity, implemented as a gradient of metabolically simple to complex media, would maintain higher diversity in a multispecies synthetic microbial community by promoting coexistence and more even species abundances. Using a fully factorial design of 96 resource environments, we exposed the community to two orthogonal nutrient gradients: total carbon availability and increasing resource complexity. Resource complexity was varied by increasing the proportion of carbon derived from R2A, a chemically diverse medium containing a mixture of substrates such as sugars, amino acids, and more complex compounds (e.g. starch and peptides) that differ in their accessibility. This increased substrate heterogeneity while total carbon availability was controlled independently by varying the proportion of glucose alone and R2A in the medium. Contrary to expectations, we found that total carbon availability was the primary driver of community-level regime shifts, with resource complexity exerting a secondary, modulatory effect, and that the emerging state shift pattern could be predicted from monoculture growth traits of the dominant species.

## Materials and methods

### Synthetic bacterial community

In this study, we used a modified, 16-species version of our synthetic community that has been used in various earlier studies [44–46]. All species were obtained from the HAMBI culture collection (University of Helsinki) and represent diverse taxonomic groups and ecological origins, including aquatic, soil, and host-associated environments. The community consisted of the following species: *Pseudomonas putida* (HAMBI-ID 6), *Agrobacterium tumefaciens (*105), *Comamonas testosteroni* (403), *Citrobacter koseri* (1287), *Morganella morganii* (1292), *Kluyvera intermedia* (1299), *Sphingobium yanoikuyae (1842), Sphingobacterium spiritivorum* (1896), *Aeromonas caviae* (1972), *Pseudomonas chlororaphis* (1977), *Bordetella avium* (2160), *Cupriavidus oxalaticus* (2164), *Paracoccus denitrificans* (2443), *Stenotrophomonas maltophilia* (2659), *Niabella yanshanensis* (3031), and *Microvirga lotononidis* (3237).

### Media preparation and composition

**Figure 1:**
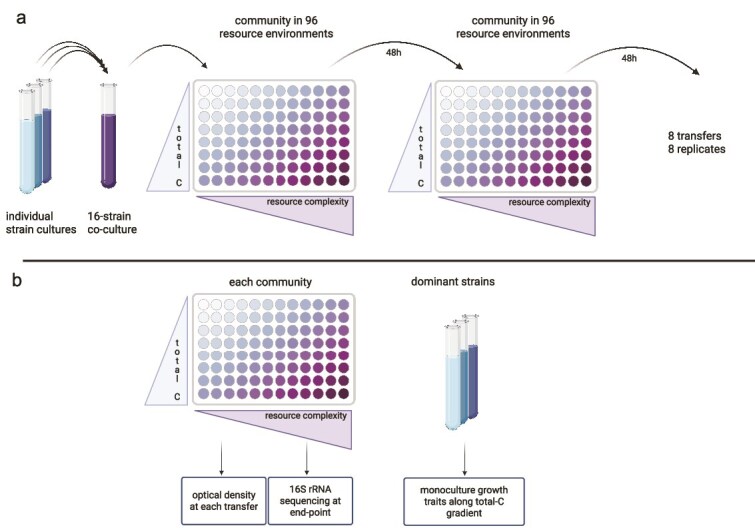
Schematic representation of experimental setup. (a) After pre-culturing each of the 16 species of the community for one growth cycle, we adjusted the optical density of each culture, pooled them at equal volume, and inoculated 96 resource environments, with each condition replicated 8 times. Each culture was serially transferred for 16 days, with 1% of the culture transferred every 48 h. (b) At each transfer, we measured OD_600_ of each community. In addition, we conducted 16S rRNA sequencing of all communities at the endpoint and measured growth traits of each of the dominant species (*A. caviae, P. chlororaphis, C. koseri*) in monoculture in all carbon levels in the absence of R2A.

For the serial passage experiment (Fig. 1), we prepared a total of 96 distinct growth media that varied along two experimental gradients: total carbon concentration and resource complexity (% R2A carbon contribution).

First, a glucose stock solution was prepared by dissolving glucose in deionized water, followed by filter sterilization. M9 minimal medium was prepared according to standard protocols by dissolving 11.28 g of M9 minimal medium salts (MP Biomedicals, UK) in 1.0 l of Milli-Q purified water and supplementing with MgSO₄ and CaCl₂. Glucose minimal media were prepared by adding glucose stock to M9 minimal medium to obtain a maximum carbon concentration of 4 g C l^−1^. R2A medium was prepared by dissolving R2A Broth powder (Neogen, UK) in M9 minimal medium. According to the manufacturer’s formulation, R2A contains a mixture of carbon sources including glucose, soluble starch, sodium pyruvate, and complex substrates from yeast extract, meat peptone, and casamino acids, supporting the growth of diverse bacteria in water samples. These provide a heterogeneous resource environment with both readily utilizable compounds (e.g. glucose, amino acids) and more complex substrates such as starch requiring extracellular degradation.

The total carbon content of R2A powder was determined at Lammi Biological Station (Finland) using a Shimadzu TOC-L csh analyzer (SFS-EN 1484), showing that R2A contained 272 mg C per gram of dry powder. Based on this measurement, R2A was added to obtain a maximum concentration of 4 g C l^−1^, corresponding to 14.7 g R2A powder per liter. Glucose minimal medium (4 g C l^−1^) and R2A medium (4 g C l^−1^) were mixed in defined proportions to generate 12 media in which the proportion of R2A-derived carbon ranged from 0 to 100% of total carbon (resource complexity gradient), while keeping total carbon constant at 4 g C l^−1^. Each of these 12 media was then diluted 1:2 with carbon-free M9 minimal medium in a serial dilution series to generate eight total carbon concentrations ranging from 0.031 to 4 g C l^−1^. This resulted in a total of 96 distinct media compositions, which are listed in Table S1.

For each condition, 1000 μl of medium was dispensed into 96-well deep-well plates (Sarstedt, Germany). Each of the 96 growth media compositions was replicated eight times.

### Pre-culture and community assembly

All 16 bacterial species were stored at −80°C in glycerol stocks. Each species was grown separately from frozen stocks in 25 ml microcosm flasks containing 6 ml of nutrient-rich proteose peptone yeast extract medium (PPY; 20 g proteose peptone and 2.5 g yeast extract per 1 l of deionized water). Cultures were incubated for 48 h at 30°C with constant shaking at 70 rpm. After incubation, cultures were washed twice with unsupplemented M9 medium, and the optical density at 600 nm (OD_600_) was adjusted to 1.4 using unsupplemented M9 medium. Equal volumes of each adjusted culture were pooled to form the synthetic community. One milliliter of the pooled community was stored at −20°C as a baseline control for amplicon sequencing.

### Serial passage experiment

We used serial transfer and culture protocols similar to those used in earlier studies [44, 45] in a serial passage experiment conducted over 16 days and consisting of eight growth cycles. Every 48 h, 1% of each culture from the previous plates was transferred into new 96-well deep-well plates containing 1 ml of fresh growth medium with the same R2A and glucose-derived carbon concentrations. At each transfer, OD_600_ was measured to monitor bacterial growth. After measurement, samples were frozen at −20°C for subsequent amplicon sequencing.

### 16S rRNA gene amplicon library preparation and sequencing

Microbial community composition was characterized using 16S rRNA gene amplicon sequencing with a two-step PCR and dual-indexing strategy. Amplicon libraries were prepared using an in-house protocol (see Supplementary methods), based on the TaggiMatrix (Adapterama II) method of Glenn *et al.* [47].

Relative abundances of the 16 bacterial species were determined by mapping paired-end 16S rRNA amplicon reads (from whole-genome sequences [48]) following the analysis workflow described by Cairns *et al.* [44]. Reads were quality-trimmed, merged, filtered, and mapped to reference 16S rRNA gene sequences for each species. Species-specific read counts were normalized according to 16S rRNA gene copy number.

### Growth assays across a glucose-derived carbon gradient

To test whether growth traits could explain the dominance shifts observed in the community experiment, we measured growth of ancestral bacterial strains for the three species that dominated in the community experiment (see Results section) across a glucose-derived carbon gradient, without R2A: *A. caviae* (1972), *C. koseri* (1287), and *P. chlororaphis* (1977). Bacteria were grown individually in 6 ml PPY medium in 25 ml microcosm flasks at 30°C with constant shaking at 70 rpm for 48 h. After incubation, cultures were centrifuged and washed twice with an equal volume of unsupplemented M9 medium and subsequently diluted 1:10 000 for inoculation. Sixteen glucose solutions with carbon concentrations ranging from 0.07–2 g C l^−1^ (each at 80% of the previous concentration; see Table S2) were prepared in M9 minimal medium. For each condition, 140 μl of medium was dispensed into 96-well plates.

Ten microliters of each diluted bacterial suspension were added to the corresponding wells of 96-well plates, with six replicates per species at each carbon concentration. Plates were sealed with breathable membranes and incubated in LogPhase600 (Agilent BioTek, USA) devices at 30°C with shaking at 800 rpm. OD_600_ was measured every 10 min for 48 h. Three serial transfers were performed by transferring 10 μl of culture into fresh plates containing the same carbon conditions at each 48 h interval.

### pH measurements

To assess whether pH changes could influence community composition, we measured pH in both the 16-species synthetic community and individual species.

For the community experiment, the 16-species community (ancestral strains) was grown in M9 minimal medium supplemented with glucose at three carbon concentrations (0.03125, 0.25, and 4 g C l^−1^). Each condition was replicated three times, with uninoculated media included as controls. A glucose stock solution was prepared in deionized water and filter-sterilized. The highest carbon medium (4 g C l^−1^, corresponding to 10 g glucose l^−1^) was prepared by dissolving the sterile glucose stock solution in M9 minimal medium supplemented with MgSO₄ and CaCl₂, and lower carbon concentrations were generated by serial dilution (1:16 and 1:8).

The community inoculum was constructed from individual ancestral strains. Each strain was first grown in 6 ml 100% PPY medium in 25 ml microcosm flasks at 30°C with constant shaking at 70 rpm for 48 h. Then, 100 μl of each culture was transferred to 6 ml of 100% R2A medium and incubated for 24 h at 30°C. Equal volumes of the R2A pre-cultures were centrifuged and resuspended in unsupplemented M9 medium, combined in equal volumes, and mixed 1:1 with 85% glycerol to generate a frozen stock stored at −80°C.

For the pH experiment, 50 μl of this stock solution was inoculated into two 25 ml microcosm flasks containing 6 ml of 100% R2A medium and incubated for 24 h at 30°C with shaking (70 rpm). OD_600_ was measured, after which 5 ml of each pre-culture was centrifuged at 3000× *g* for 3 min. The supernatant was removed, and pellets were resuspended in 4 ml of unsupplemented M9 medium before combining the cultures. OD_600_ was measured again, and 500 μl of the combined community was inoculated into Erlenmeyer flasks containing 50 ml of M9 minimal medium supplemented with glucose at the three carbon concentrations. Cultures were incubated at 30°C for 48 h. The pH of each medium was measured immediately after inoculation and again after incubation using a calibrated pH meter. The pH of the glucose stock solution and uninoculated media at each carbon concentration was measured as baseline control values.

For individual species, all 16 strains were grown separately in M9 minimal medium supplemented with glucose (4 g C l^−1^). Pre-cultures were prepared by retrieving each species from frozen glycerol stocks and growing them in 6 ml of 100% R2A medium in 25 ml microcosm flasks at 30°C for 2 h with shaking (70 rpm). OD_600_ was measured after pre-culture growth and again after washing. One milliliter of each pre-culture was centrifuged at 3000× *g* for 3 min, the supernatant removed, and the pellet resuspended in 1 ml of unsupplemented M9 medium. Subsequently, 100 μl of the washed culture was inoculated into 10 ml of M9 minimal medium supplemented with glucose (4 g C l^−1^). Cultures were incubated at 30°C for 48 h with shaking (70 rpm), and OD_600_ and pH were measured after incubation (Fig. S1). Uninoculated medium was used as a baseline control.

### Statistical analyses

All statistical analyses were conducted in R version 4.4.2 [49]. Alpha and beta diversity metrics across analyses were computed using the vegan package [50].

#### Community composition

To quantify the effects of resource gradients on community composition, we calculated Bray–Curtis dissimilarities from relative abundance matrices and tested for marginal effects of total-C and resource complexity using permutational multivariate analysis of variance (PERMANOVA; 9999 permutations), as implemented in the vegan package [50, 51]. Total carbon concentration and resource complexity (% R2A carbon contribution) were included as continuous predictors, and marginal sums of squares were used to assess their independent contributions to compositional variance.

Low-dimensional ordination of communities was performed using *t*-distributed stochastic neighbor embedding (*t*-SNE) with the Rtsne package on the relative abundance matrix, using PCA-initialized inputs, perplexity = 35, θ = 0.5, and 7000 iterations [52]. To identify discrete compositional states, density-based clustering was applied to the two-dimensional *t*-SNE embedding using DBSCAN (eps = 4, minPts = 4; dbscan package), with noise points excluded from cluster assignment. To assess the role of the simple versus complex resource gradients in structuring community states, *t*-SNE ordinations were visualized globally and faceted by total-C concentration. Community repeatability across replicates was quantified as dispersion around group centroids in ordination space, using the betadisper function in the vegan package, with higher dispersion interpreted as lower compositional repeatability.

#### Monoculture growth traits

We fit linear models using lag time, maximum growth rate, and area under the curve (AUC) as response variables and species identity, total-C level, and their interaction as explanatory variables, performing ANOVA tests on the models. For pairwise comparisons, we calculated estimated marginal means (EMMs) with the emmeans package. [53]

To test whether monoculture traits predict community dominance across strains, we modeled strain relative abundance as a function of standardized lag time, maximum growth rate, and AUC, including strain identity as a covariate. For carbon-level aware analysis, the dominant species abundance data from the community experiment in the glucose-alone axis were linked to monoculture trait data at the nearest-matching glucose levels. Analyses were performed separately for the full carbon gradient and for the intermediate transition zone (0.25–1.0 g l^−1^). We quantified model fit using *R^2^* and estimated the unique contribution of each trait as the change in *R^2^* upon its removal from the full model. For comparison, we also calculated marginal *R^2^* values from univariate regressions of relative abundance on each trait.

#### Biomass and diversity

Community biomass (OD_600_) was analyzed with linear models including total-C concentration, resource complexity, and their interaction. Model terms were evaluated using ANOVA on the fitted models, and variance explained was summarized using model *R^2^* and an additive-vs-interaction model comparison.

To characterize how diversity responded to the two resource axes, Shannon diversity was quantified in two complementary ways. First, linear models of Shannon diversity included total carbon, resource complexity, and their interaction. Term significance was obtained from ANOVA, and variance contributions were decomposed by comparing *R^2^* from nested models. Second, the non-linear “hump” response along each resource axis was quantified using a conservative peak-height metric computed on observed means (peak at interior levels relative to both endpoints), with 95% confidence intervals obtained by bootstrap resampling observations within each curve (i.e. within each fixed level of the other resource).

Compositional variability, computed as described above with vegan as the mean Bray–Curtis distance to the group centroid, was correlated with mean Shannon diversity per cell in the resource grid using Pearson and Spearman correlation tests. The associated scatterplot used an ordinary least-squares fit with 95% confidence bands for visualization.

Portions of the analysis code were refactored with the assistance of ChatGPT (GPT-5.2 Thinking; OpenAI, accessed February 2026). All code and outputs were verified by the authors.

## Results

### Level of total carbon availability drives community dynamics

**Figure 2:**
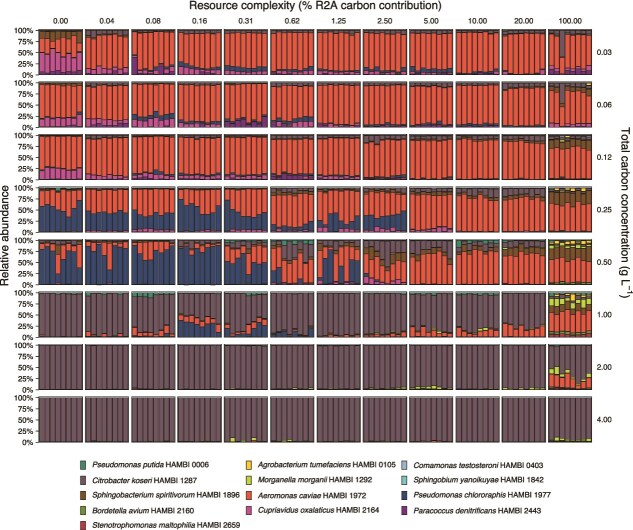
Relative abundance of species in community in all resource environments after 16 day serial passage experiment. Each bar chart represents the composition of a serially transferred microbial community in relative abundances after 16 days of passaging in the 96 different resource environments. The total carbon gradient is represented on the *y*-axis, and the resource complexity gradient is represented on the *x*-axis. The eight adjacent bar charts within each unique treatment combination represent replicate communities. The figure contains 768 data points (8 replicates in 96 resources).

Serially transferring our 16-species community for 16 days in 96 resource environments (8 carbon levels × 12 complexity levels) and using an in-house, previously undescribed amplicon library preparation protocol (based on the TaggiMatrix (Adapterama II) method of Glenn *et al.* [47], see Supplementary methods, primers are listed in Supplementary Tables S12 and S13), we observed that both resource dimensions significantly influenced community composition; however, variation along the total carbon availability (total-C) gradient explained substantially more compositional variation than along the resource complexity (RC) gradient (PERMANOVA, marginal effects: total-C *F*(1,765) = 968.9, *R^2^* = 0.546, *P* = .0001; RC *F*(1,765) = 39.5, *R^2^* = 0.022, *P* = .0001; Table S3; Fig. 2). While numerous lower-abundance taxa exhibited gradual changes in frequency across both gradients (Fig. S2), overall community dynamics were dominated by three species, whose relative abundances shifted sharply along the total carbon axis (Fig. 2). At the low-total-C end of the gradient (0.03125 g C l^−1^, 0.0625 g C l^−1^, 0.0125 g C l^−1^), *A. caviae* reached the highest abundance, while at high total-C (1 g C l^−1^, 2 g C l^−1^, 4 g C l^−1^) *C. koseri* dominated community composition. In the intermediate zone (0.25 g C l^−1^, 0.5 g C l^−1^) *P. chlororaphis* rose to high abundance, alongside *A. caviae*. Additional shifts in lower-abundance taxa were not uniform but showed structured variation across the resource grid: for example, certain taxa increased specifically under low total-C and low complexity conditions, whereas others became more prominent at higher resource complexity and intermediate-to-high (but not maximal) total-C levels (Fig. 2). This indicates that, alongside the dominant species–driven state transitions, finer compositional gradients and niche differentiation occurred among less abundant members of the community.

We found that the change in key species along the total-C gradient was not driven by changes in community biomass (Fig. S3). Instead, the same three-state community pattern emerged with absolute abundance (relative abundance related to community biomass) as with relative abundance. In other words, major increases and decreases in the relative abundance of the three dominant species were accompanied by corresponding shifts in their absolute abundances despite strong increases in community biomass at increasing total-C levels.

**Figure 3:**
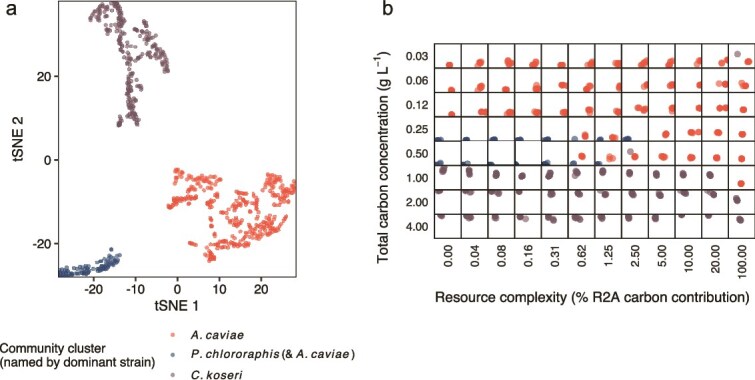
*t*-SNE visualization (ordination) of 16S rRNA data of community after 16 day serial passage experiment. (a) Global clustering of community into three states. These correspond to communities dominated by *A. caviae*, co-dominated by *P. chlororaphis* and *A. caviae*, or dominated by *C. koseri*. The figure contains 768 data points (8 replicates in 96 resources). (b) Same ordination partitioned by resource levels.

Ordination analysis revealed that communities clustered into three major compositional states corresponding to dominance by *A. caviae*, co-dominance by *P. chlororaphis* and *A. caviae*, or dominance by *C. koseri* (Fig. 3a). These clusters were primarily structured by the total-C gradient, reflecting discrete switches in dominance among the three species. Partitioning the same ordination by resource levels showed that resource complexity modulated these primary dynamics: the co-dominant *P. chlororaphis–A. caviae* state occurred only at low levels of resource complexity, whereas high resource complexity favored exclusive dominance states (Fig. 3b). Square-root transforming the relative abundance data to give more weight to low-abundance species added more granularity to the community structures in the resource grid (Fig. S4) but did not alter this three-state pattern governed by three dominant species beyond substructural changes (Fig. S5).

Notably, lowest repeatability between the replicate communities (highest community divergence from group centroid) was observed in the transition zone of the three states (Fig. S6).

In summary, we observed a repeatable state shift pattern in the serially transferred multispecies synthetic community. The dominant species in the three clusters (*A. caviae*—low total-C, *P. chlororaphis*—intermediate total-C, *C. koseri*—high total-C) are generalists regarding resource metabolism [44, 45]. Together, these results indicate that total carbon availability was the principal driver of community-level state transitions, with resource complexity exerting a secondary, modulatory effect.

### Monoculture lag phase duration explains community outcome

**Figure 4:**
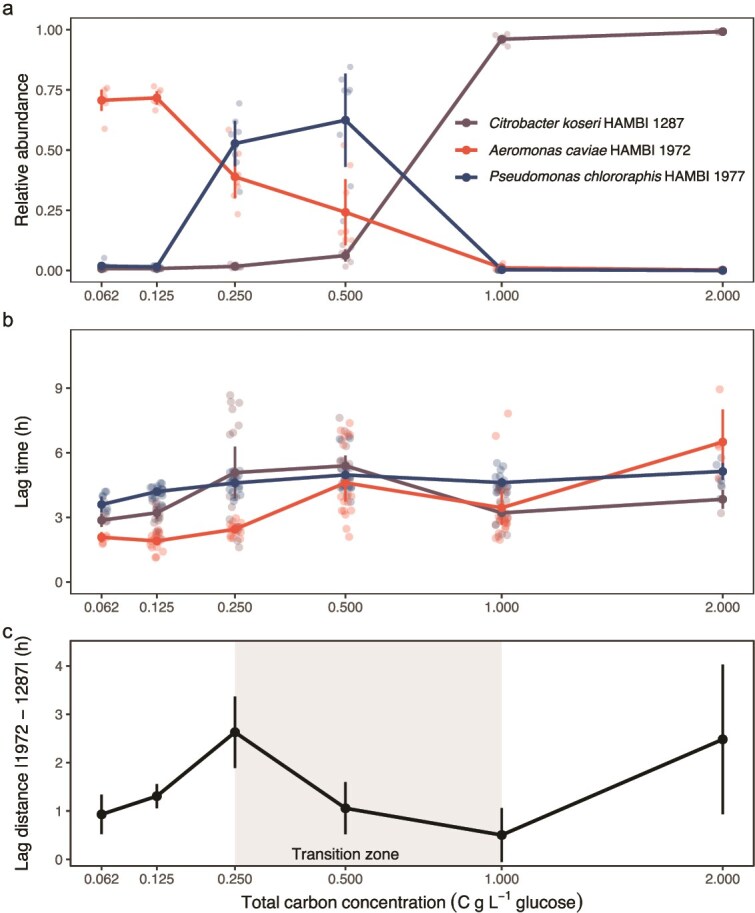
Three-panel figure detailing monoculture growth traits of three dominant species along carbon gradient (mean ± 95% confidence intervals). (a) Relative abundance of species (*N* = 8 replicates per species per time point). (b) Duration of lag time of each species at matching carbon levels (*N* = 12–18 per species per time point). (c) Distance between lag phase duration between the two most abundant species.

Having observed the partitioning of the community composition into the three clusters along the total-C gradient, we next examined factors that could explain the primary dynamics of the three dominant species (*A. caviae, C. koseri, P. chlororaphis*; Fig. 4a). We cultured the most abundant species of the community experiment at each total-C level but without added complexity, performing three serial transfers to acclimate species to each resource level. In monocultures, these three species differed significantly in lag time in a total-C-dependent manner (Fig. 4b; linear model, species × total-C interaction: *F*(2, 42) = 9.42, *P* < .001), but not in maximum growth rate, carrying capacity, or area under the growth curve (AUC; species × total-C interactions: *F*(2, 42) = 1.50, *P* = .236; *F*(2, 42) = 0.46, *P* = .64; and *F*(2, 42) = 1.25, *P* = .30, respectively; Table S4). Species also differed in maximum growth rate and AUC in a total-C-independent manner (species main effects: *F*(2, 44) = 19.66, *P* < .001 and *F*(2, 44) = 3.43, *P* = .041, respectively), but not in lag time or carrying capacity (species main effects: *F*(2, 44) = 0.86, *P* = .43 and *F*(2, 44) = 2.47, *P* = .096, respectively). Maximum growth rate ranked consistently across total-C levels as *P. chlororaphis > A. caviae > C. koseri* (Tukey-adjusted pairwise contrasts, all *P* < .02; Table S5). AUC showed the same species ordering, but differences were weaker and did not reach significance in pairwise comparisons (Table S6). However, this ranking was inconsistent with community outcomes, where mostly *A. caviae* or *C. koseri* dominated depending strongly on total-C level. These results indicate that lag time was particularly influenced by total-C level in monocultures.

We next tested whether monoculture growth traits predicted community dominance across total-C levels. Across the total-C gradient, shorter lag time was the strongest independent predictor of the abundance of the three dominant species (unique *R^2^* = 0.314, *P* < .001), explaining over twice as much additional variance as higher maximum growth rate (unique *R^2^* = 0.136, *P* < .001), while higher AUC contributed little (unique *R^2^ =* 0.039; Table S7). In the intermediate species transition zone (0.25–1.0 g C l^−1^), lag remained the single strongest independent predictor (unique *R^2^* = 0.439, *P* < .001), but maximum growth rate contributed nearly as much additional variance (unique *R^2^* = 0.349), indicating that both traits shaped dominance under these conditions (Fig. 4c).

When considered alone, lag explained less variation in the transition zone (*R^2^* = 0.112) than across the full gradient (*R^2^* = 0.161), consistent with reduced divergence in lag times (Fig. 4b). In contrast, the standalone predictive power of growth rate increased in the transition zone (*R^2^* = 0.083 vs 0.018). Thus, while lag remained central to competitive success, its dominance as the primary predictor weakened as lag times converged and growth rate became comparatively more influential.

Overall, monoculture growth traits had a moderate ability to quantitatively predict the major shifts in community dominance across the total-C gradient, with lag time structuring outcomes at carbon extremes and growth rate becoming comparably important as lag divergence decreased.

### Relationship between total carbon level and diversity depends on resource complexity

Both gradients (total-C, resource complexity) showed strong, positive, and approximately linear relationships with community biomass (Fig. S7). Biomass was dominated by total-C availability, with smaller but significant contributions from resource complexity and their interaction (linear model: total-C *F*(1,764) = 878.7, *P* < .001; RC *F*(1,764) = 51.8, *P* < .001; total-C × RC *F*(1,764) = 102.3, *P* < .001), together explaining 57.1% of the variance in biomass (Tables S8 and S9).

**Figure 5:**
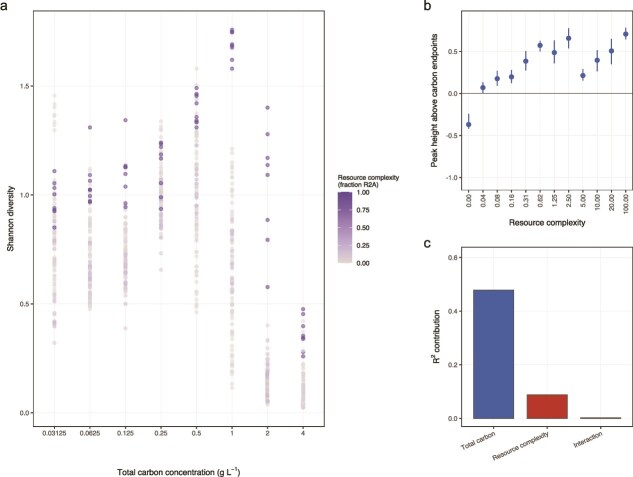
Diversity response along resource gradients. (a) Shannon diversity shown separately for each resource environment. The *x-*axis shows the total carbon level (doubling concentrations spaced evenly) and point color indicates resource complexity level. The figure contains 768 data points (8 replicates in 96 resources). (b) Hump metric for total carbon gradient (*y*-axis) at each level of resource complexity (*x*-axis). Points show the estimated peak height (hump statistic); error bars show 95% bootstrap confidence intervals. (c) Variance partitioning of Shannon diversity using nested linear models. Bars show unique *R^2^* contributions of total carbon level, resource complexity (% R2A carbon contribution), and their interaction.

In contrast, Shannon diversity responded to the two resource axes in qualitatively different ways (Fig. 5). Along the total-C gradient, diversity declined sharply at high concentrations across all levels of resource complexity (Fig. 5a), reflecting dominance by *C. koseri*, with a peak at intermediate total-C levels. Along the resource complexity gradient, diversity was comparatively stable across most levels and increased only at the highest complexity level. The non-linear diversity response along the total-C gradient was quantified using a conservative hump metric (Fig. 5b), which was positive with 95% bootstrap confidence intervals excluding zero across all RC levels except 0%, confirming consistent peak diversity at intermediate total-C concentrations. Thus, total-C primarily imposed a non-linear constraint on diversity, whereas resources complexity buffered diversity across a broad range of levels.

These patterns were reflected in statistical models: total-C explained the largest share of variation in Shannon diversity (linear *model: F*(1,765) = 846.0*, P* < .001; *R^2^* = 0.479), followed by a smaller but significant effect of resource complexity (*F*(1,765) = 156.0, *P* < .001; *R^2^* = 0.088; Fig. 5c; Tables S10 and S11). The total-C × RC interaction was statistically significant but accounted for little additional variance (*F*(1,764) = 4.15, *P* = .042; *R^2^* = 0.002), indicating that the two resources act largely independently.

Furthermore, mean Shannon diversity was strongly correlated with community dispersion across the resource grid (Pearson *r* = 0.67, Spearman *ρ* = 0.82, both *P* < .001; Figs. S8 and S9), indicating that conditions supporting higher among-replicate compositional variability (transition zone between dominant species regimes; Figs 2 and 3) also exhibited greater within-community diversity.

### Carbon-dependent acidification is linked to microbial productivity rather than species identity

In the 16-species community, pH decreased with increasing carbon concentration, consistent with greater accumulation of acidic metabolic byproducts at higher resource levels. However, this pattern coincided with increased culture density, making it difficult to distinguish direct effects of carbon concentration from those mediated by microbial productivity. To disentangle these effects, we examined pH changes in individual species grown at a fixed high carbon concentration (4 g C L^−1^). Across species, final pH was negatively associated with culture density (OD_600_), indicating that acidification increases with microbial growth (Fig. S1). However, species differed in the magnitude of pH change, with some taxa (e.g. *P. chlororaphis*) reducing pH more strongly than others (e.g. *C. koseri*) at comparable culture densities.

Together, these results show that pH shifts occur along the carbon gradient and reflect both overall microbial productivity and species-specific metabolic differences. Thus, while pH changes could contribute to community dynamics, they are closely linked to variation in microbial growth and cannot be attributed solely to the presence of particular acidifying species.

## Discussion

We serially transferred a 16-species synthetic microbial community across a broad range of resource environments for 16 days and consistently observed a state shift pattern along the total-C gradient. Thus, total-C availability was the principal driver of community-level regime shifts. This is supported by the strong positive relationship between total-C and community biomass and the non-linear impact of total-C on diversity, which declined at high concentrations and peaked at intermediate levels. Resource complexity had a smaller but significant effect, particularly by buffering diversity across a broad range of total-C levels. Both resource gradients acted independently in shaping community composition and diversity, with their interaction explaining little additional variance.

Along the total-C gradient we observed discrete shifts between three dominant community states, accompanied by increasing biomass and a non-linear diversity response. Resource quantity therefore simultaneously intensifies productivity while constraining diversity, revealing a complex relationship between resource supply and community structure [8]. Glucose, the principal carbon source in the total-C gradient, likely represents a key niche axis for dominant species (*A. caviae, P. chlororaphis*, and *C. koseri*), whereas increasing proportions of R2A-derived carbon buffer diversity without fundamentally restructuring community states. The highest-diversity communities occurred where single-species dominance was weakest. In particular, diversity peaked at intermediate total-C and at high resource complexity combined with high (but not maximal) total-C, indicating that multiple compositional configurations can support elevated diversity.

These findings align with ecological theory predicting that resource gradients can drive community shifts through competitive exclusion or coexistence [9]. They also support the framework of Gonze *et al.* [54], in which alternative community compositions arise in response to gradual environmental change. The state shift pattern emerged along a continuous carbon gradient, indicating that changes in resource supply alone can reorganize community states without altering the species pool or colonization history. At the same time, the clustering of communities into discrete compositional types mirrors previous observations that microbial communities often occupy distinct states rather than forming smooth continua [28, 30, 55]. Our data suggest that such clustering arises from competitive dynamics among a few dominant taxa interacting with resource supply.

The broader literature contextualizes these patterns. Dal Bello *et al.* [17] showed that increasing resource complexity can increase bacterial richness, though often less than predicted by niche-expansion models, while Goldford *et al.* [15] demonstrated that carbon source identity strongly determines community composition at higher taxonomic levels. Together with our results, this suggests that resource quantity structures primary community states whereas resource complexity modulates diversity within them. Finally, the partial predictability of dominance shifts from monoculture growth traits indicates that species-level life-history differences can retain a measurable imprint on competitive sorting even in multispecies communities where cross-feeding and environmental modification may occur [56].

To mechanistically probe the dominant role of total carbon level, we performed monoculture-community analysis on the glucose-only total-C axis, which isolates carbon availability in the absence of resource complexity. Monoculture growth traits provided a mechanistic link between individual performance and community dominance, but their importance depended on carbon availability. Lag phase and maximal growth rate are known to influence microbial competition [24], yet their predictive value proved environmentally contingent. Lag phase can generate strong priority effects in serial-transfer systems, where early starters gain disproportionate access to resources [25]. Under carbon-poor conditions, these effects are likely amplified because early growth rapidly exhausts limited resources, allowing small differences in lag to determine competitive outcomes.

Although the three dominant strains (*A. caviae, P. chlororaphis*, and *C. koseri*) are metabolic generalists [45], they likely differ in substrate preferences [57, 58] and broadly reflect contrasting ecological strategies. *Aeromonas* species often persist in resource-scarce aquatic habitats [59–63], consistent with an oligotrophic tendency [64, 65], whereas *C. koseri*, a close relative of *Escherichia coli* [66], reflects a more copiotrophic strategy [64, 65]. Such differentiation may maintain pronounced lag divergence at carbon extremes where early-start advantages dominate. At intermediate carbon supply, where lag differences diminish, competitive sorting shifts toward maximum growth rate as part of overall growth performance [24]. Together these results indicate that carbon availability structures community transitions by modulating which life-history trait governs dominance: lag phase dominates when timing differences persist, whereas growth rate becomes more important as lag divergence decreases.

Furthermore, our findings highlight the differential effects of resource complexity on diversity and composition. While the total-C gradient imposed a non-linear constraint on diversity, with a peak at intermediate concentrations, resource complexity increased diversity within each total-C level, thereby buffering communities against the diversity-reducing effects of low or high carbon availability. This suggests that resource complexity can stabilize microbial communities, particularly where simple resources are limiting or variable [16, 67]. The stress gradient hypothesis (SGH), originally developed in plant ecology, proposes that environmental stress modulates species interactions, shifting from facilitation under low stress to competition under high stress [32, 33]. Consistent with this framework, *C. koseri* competitively excluded most other species in the high total-C, low-diversity cluster. However, this pattern applied only to the total-C gradient and not to increasing resource complexity, likely because higher complexity maintains diversity by alleviating direct competition.

As a constraint of the study, we note that the relative strength of total-C and resource complexity effects can depend on how these gradients are represented experimentally. In our system, resource complexity was implemented by controlling for the carbon contribution from glucose alone versus from R2A. Readily available carbon sources in R2A include glucose and amino acids, and it is unclear how readily available more complex substrates such as starch are. The number of readily available resources influences the effective complexity of the resource. A low effective resource complexity could explain the dominance of total-C versus resource complexity in our study. In natural communities, complex resources typically include resources with varying degrees of availability, making our setup relevant even in the case of a low effective resource complexity. However, future studies could explicitly address this issue by designing complex resources based on knowledge of resource availability.

In conclusion, our results show that, within the range of resource conditions tested here, total carbon availability structures microbial community states, while resource complexity primarily modulates diversity within those states. These findings highlight the central role of resource quantity in shaping community-level organization and underscore how different resource dimensions interact to govern diversity patterns. Understanding how resource gradients shape community organization will help develop more realistic predictive models of microbial ecosystem functioning.

## Supplementary Material

Supplementary_material_ycag149

## Data Availability

The 16S rRNA amplicon sequencing data generated in this study have been deposited in the European Nucleotide Archive (ENA) under accession number PRJEB108454. All processed data tables and analysis outputs supporting this study are publicly available on Zenodo at https://zenodo.org/records/20019522 (DOI: 10.5281/zenodo.20019521). The repository includes community composition tables derived from 16S rRNA amplicon sequencing, optical density measurements across the resource grid, monoculture growth trait summaries, and strain-level pH and OD_600_ measurements.
